# Immune Responses to RNA Viruses

**DOI:** 10.1155/2018/5473678

**Published:** 2018-06-12

**Authors:** Elias A. Said, Felipe Diaz-Griffero, Dorine Bonte, Daniel Lamarre, Ali A. Al-Jabri

**Affiliations:** ^1^Department of Microbiology and Immunology, College of Medicine and Health Sciences, Sultan Qaboos University, P.O. Box: 35, 123 Muscat, Oman; ^2^Department of Microbiology and Immunology, Albert Einstein College of Medicine, New York City, NY, USA; ^3^CNRS UMR8200, Gustave Roussy Cancer Institute, Paris, France; ^4^College of Pharmacy, Paris-Sud University, Paris, France; ^5^Centre de Recherche du CHUM (CRCHUM) et Faculté de Médecine, Université de Montréal, Montréal, QC, Canada

RNA viruses constitute an important threat to human health around the globe. Several RNA viruses are pandemic and infect hundreds of millions around the world leading to the death of millions of people every year. These viruses include the human immunodeficiency virus (HIV), hepatitis C virus (HCV), Ebola virus, Zika virus, respiratory syncytial virus (RSV), influenza viruses, yellow fever virus, dengue virus, rhinoviruses (common cold), human T-lymphotropic virus type 1 (HTLV-I), poliovirus, and measles virus. Currently, no vaccine or specific treatment is available for many of these viruses and some of the available vaccines and treatments are not highly effective. Because infection with RNA viruses is a global issue, the Journal of Immunology Research arranged for the publication of a special issue dedicated to the topic of immune responses to RNA viruses. The special issue contains 3 reviews and 2 research articles submitted by researchers from 6 countries in Europe, Asia, America, and Africa. These articles emphasized on the importance of the relationship between the components of the immune system and the treatments and vaccines directed against RNA viruses. They also highlighted the role of the immune system in the pathogenesis of the infections with these viruses.

HIV infection is a good example of the interactions between an RNA virus and the immune system and how these interactions modulate the pathophysiology of the disease caused by the virus. In this special issue, 3 articles focused on HIV infection. I. E. Akase et al. showed in “Immune Dysfunction in HIV: A Possible Role for Pro- and Anti-Inflammatory Cytokines in HIV Staging” that the levels of proinflammatory cytokines IL-6 and IL-10 are elevated in the absence of anti-HIV treatment. These cytokines, particularly IL-6, were associated with the WHO clinical staging of the disease. A trend of association was also found between these cytokines and the CD4 T cell count in HIV-infected patients. Moreover, L. Herráiz-Nicuesa et al. reported in their article entitled “Impact of the Polymorphism *rs9264942* near the HLA-C Gene on HIV-1 DNA Reservoirs in Asymptomatic Chronically Infected Patients Initiating Antiviral Therapy” that the −35 C allele is associated with low viremia and low levels of HIV reservoir. However, these associations did not reach statistical significance after thirty-six months of therapy. In addition, G. V. Gonzalez-Enriquez et al. reviewed in “SERINC as a Restriction Factor to Inhibit Viral Infectivity and the Interaction with HIV” the capacity of some HIV proteins, that is, Nef, Env, and glycosylated Gag (glycoGag), to interfere with the activity of serine incorporator 5 (SERINC5) restriction factor that prevents fusion and hence inhibits viral infectivity. The authors proposed that SERINC5 can be considered as an element in “promising scenarios” aiming to develop anti-HIV treatments and to predict the prognosis of the disease. Additionally, in their review “Viruses Seen by Our Cells: The Role of Viral RNA Sensors,” E. A. Said et al. discussed the implication of Toll-like receptors 3, 7, and 8 (TLRs 3, 7, and 8) in mounting proper anti-HIV responses and they reviewed the possibilities of targeting these molecules in future anti-HIV therapies and vaccine preparations.

The innate immune system plays a key role in sensing RNA viruses. This has a major influence on the antiviral responses and the pathogenesis of diseases caused by these RNA viruses. In their article, E. A. Said et al. also reviewed the role of pathogen recognition receptors (PRRs) in detecting nucleic acids as pathogen-associated molecular patterns (PAMPs) present in RNA viruses and how they balance the need for innate defenses against pathogens and actively restrict involuntary pathway activation. Moreover, they described the nature of the ligands and the pathways related to classic RNA helicases of antiviral innate immunity (RIG-I, MDA5) and of sentinel sensors (LGP2, SNRNP200, and DDX60) as well as of TLRs 3, 7, and 8 upon infection. They also reported on the implication of these molecules in the pathogenesis of the diseases caused by RNA viruses and the potential therapies and vaccines that involve these PRRs. In addition, in the review “Pulmonary Susceptibility of Neonates to Respiratory Syncytial Virus Infection: A Problem of Innate Immunity?,” C. Drajac et al. questioned the role of the innate immune system in the susceptibility of neonates to the infection with RSV. They discussed the knowledge about the immune environment in the lung during the early-life periods at steady state and following RSV infection. They also reviewed the different experimental strategies that aimed to modulate the neonatal susceptibility to RSV.

Altogether, the authors of these articles highlighted the danger that infections with RNA viruses represent to global health. They provided clues about how understanding and interfering with the immune responses during these infections can be a major asset in the attempt to eradicate these viral infections. In summary, this special issue is providing information about different aspects of the immune responses during infections with RNA viruses. It is reporting on the current challenges in the field and providing new perspectives for the development of efficient treatments and vaccines directed against RNA viruses.

*Elias A. Said* *Felipe Diaz-Griffero* *Dorine Bonte* *Daniel Lamarre* *Ali A. Al-Jabri*

